# Correlation between the degree of correction of neuromuscular scoliosis and patient quality of life

**DOI:** 10.6061/clinics/2017(02)02

**Published:** 2017-02

**Authors:** David Gonçalves Nordon, Ariel Falbel Lugão, Lucas Castrillon Carmo Machado, Raphael Martus Marcon, Alexandre Fogaça Cristante, Tarcísio Eloy Pessoa de Barros Filho, Olavo Biraghi Letaif

**Affiliations:** Hospital das Clínicas da Faculdade de Medicina da Universidade de São Paulo, Instituto de Ortopedia e Traumatologia, Divisão de Cirurgia da Coluna, São Paulo/SP, Brazil

**Keywords:** Neuromuscular, Scoliosis, Surgery, Quality of Life, Questionnaire

## Abstract

**OBJECTIVE:**

There are few data on patient satisfaction with surgery for the correction of neuromuscular scoliosis or on the correlation between patient satisfaction and the degree of curve correction achieved by surgery. Our aim was to determine the correlations between both patient satisfaction and perception of quality of life and the degree of curve correction.

**METHODS:**

We interviewed 18 patients and administered a questionnaire that collected social and economic data and information about functional ability, comorbidities and satisfaction. Statistical analysis was performed using chi-square tests, Pearson correlation and paired t-tests.

**RESULTS:**

The mean correction achieved was 42.8%, i.e., 34.17 degrees. Early and late complication rates were low (11.1% each). Almost all of the patients (94.4%) were satisfied with the surgery, and expectations were met for 61.1% of them. Quality of life and aesthetics were improved in 83.4% and 94.4% of cases, respectively. No correlation was found between satisfaction and degree of correction.

**CONCLUSION:**

Our surgical results are similar to those of other studies with respect to the degree of correction and patient satisfaction. The disparity between satisfaction and fulfillment of expectations may be due to unrealistic initial expectations or misunderstanding of the objective of surgery. Our findings corroborate the hypothesis that satisfaction is multifactorial and not restricted to a quantitative goal. The satisfaction of patients who undergo operation for neuromuscular scoliosis does not depend directly on the degree of deformity correction. The relationship between satisfaction and the success of the correction procedure is complex and multifactorial.

## INTRODUCTION

Scoliosis is a disease characterized by the development of abnormal curves in the spine that are greater than 10 degrees in the coronal plane. This disease is usually associated with kyphosis, lordosis, and rotations, which develop into tridimensional deformity patterns that are considerably detrimental to the patient’s quality of life (QOL). The incidence of idiopathic scoliosis in the population is 0.47-5.2% 1; there are no precise data on the incidence of neuromuscular scoliosis due to the heterogeneity of the patients and their pathologies. Data from Canada showed an annual cost of CAD$4865 per patient for those who did not undergo corrective surgery and an annual cost of CAD$29,777 for those who were operated on. The surgery costs approximately US$30,000 to US$60,000 2,3. The social and economic impacts of this condition are thus substantial.

Neuromuscular scoliosis is secondary to other pathologies, such as muscular dystrophy, cerebral palsy, spinal amyotrophy, and diseases related to the closure of the neural tube, e.g., myelomeningocele. Each year, approximately 25,000 new cases of cerebral palsy are diagnosed, and 25-64% of cerebral palsy patients will develop some degree of scoliosis 4,5. Therefore, treatment of neuromuscular scoliosis is aimed not at curing the basic pathology but at preventing its progression. There are several types of treatment, the choice of which directly depends on the extent of skeletal maturity and the severity and stage of the disease. There are multiple treatment techniques, which include options from ortheses to a variety of surgical techniques 6-9.

Surgical treatment presents a high rate of complications (in some cases, up to 68%) 10; complications include infection, the need for surgical revision, and death. However, caretakers and patients are generally satisfied with the results of surgical treatment 11.

There are several methods for evaluating patient QOL; the most commonly used method is evaluation using the SF-36 form 12. The SRS-22 is used for QOL evaluation of patients with idiopathic scoliosis 13. However, there is no common, validated questionnaire that considers the wide range of neuromuscular scolioses. Some studies have attempted to develop their own evaluation form for neuromuscular scoliosis according to the basal pathology, such as cerebral palsy and Duchenne’s dystrophy; however, the heterogeneity of patients hinders the development of a unified questionnaire 14-17.

Although some articles have evaluated the satisfaction of patients who have undergone surgery 11, there are few data in the literature that show the relationship between the degree of correction and patient satisfaction and perception of QOL.

### Objective

This article aimed to determine the correlations of both patient satisfaction and perception of the patient/caregiver QOL with the degree of scoliosis correction.

## METHODS

### Inclusion and exclusion criteria

#### Inclusion criteria

All patients with neuromuscular scoliosis who were operated on between 2013 and the first semester of 2015 at our institution and who had not undergone prior correction were included.

Patients were operated on using a posterior approach with pedicle screws.

#### Exclusion criteria

Patients with scoliosis other than neuromuscular scoliosis were excluded, as were patients with incomplete radiographic series that compromised the calculation of the Cobb angle.

### Interview

**Figure 1:**
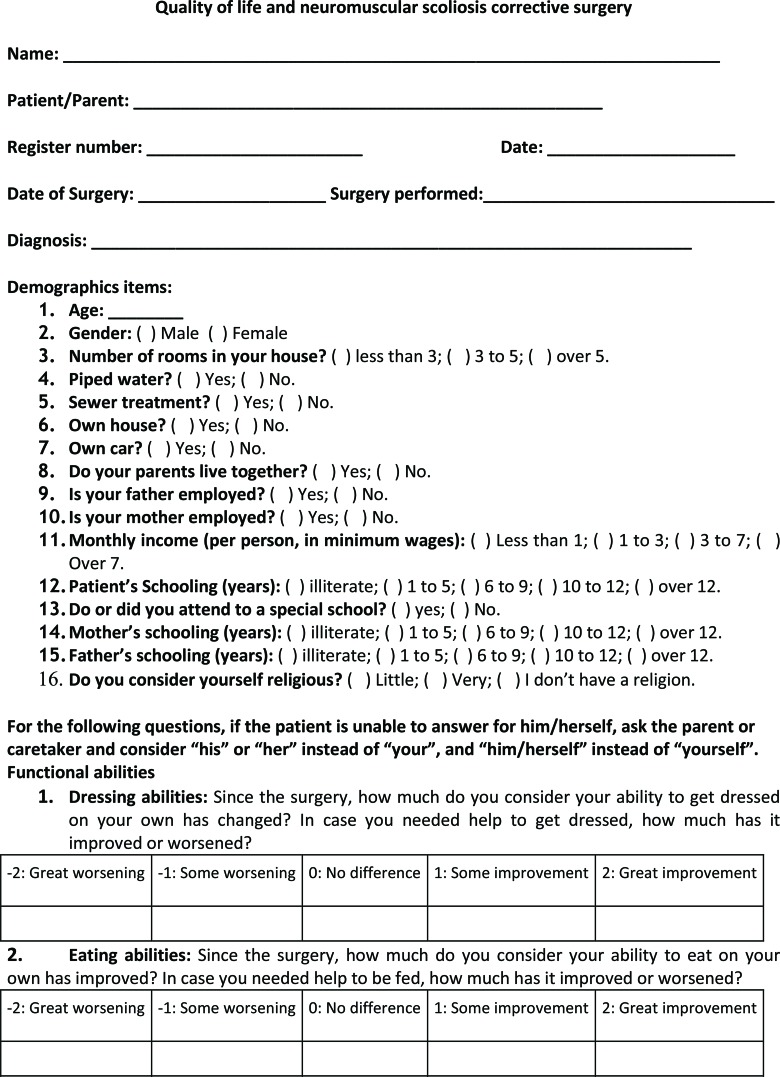

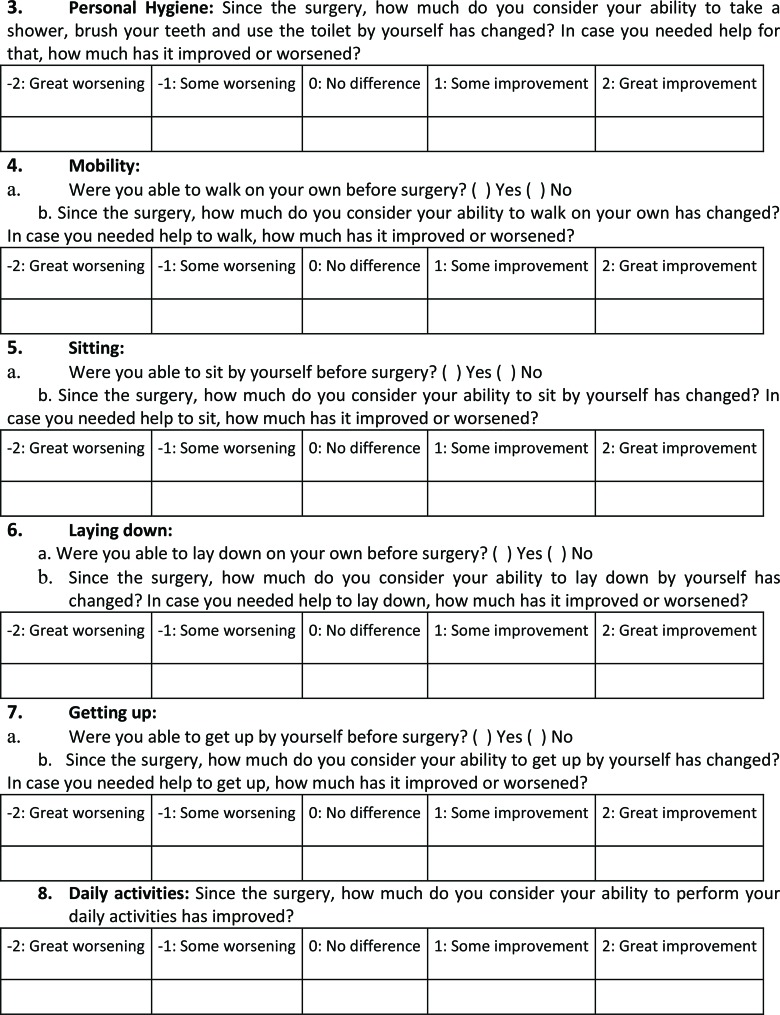

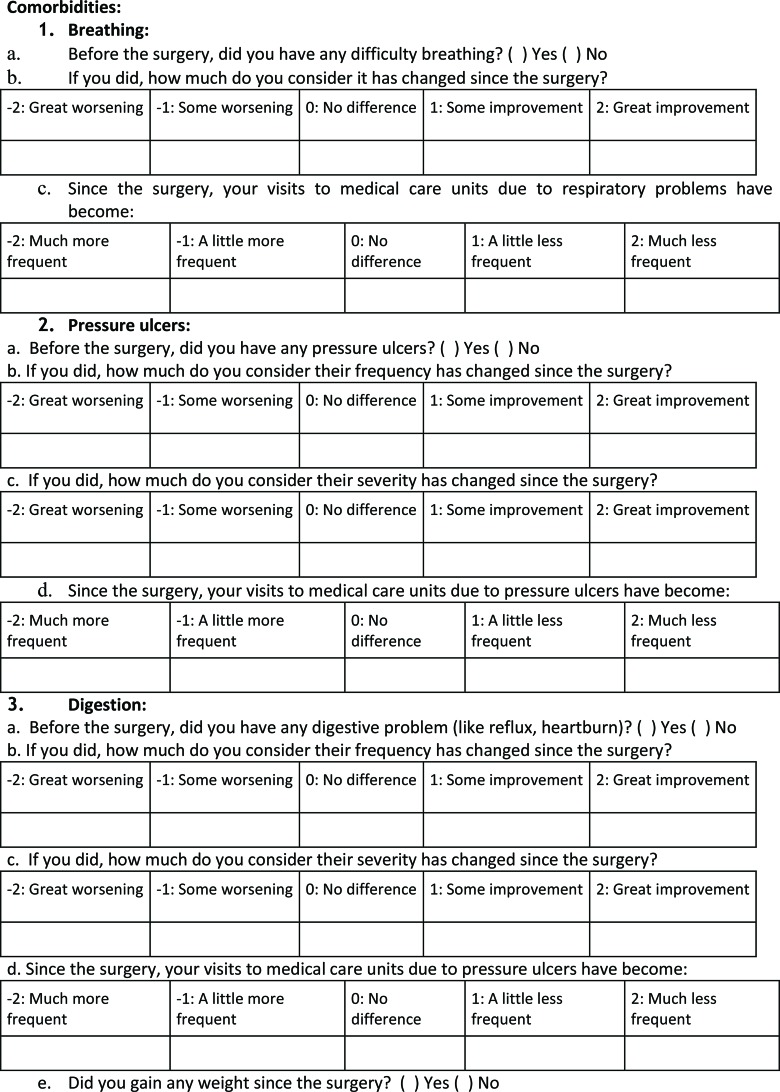

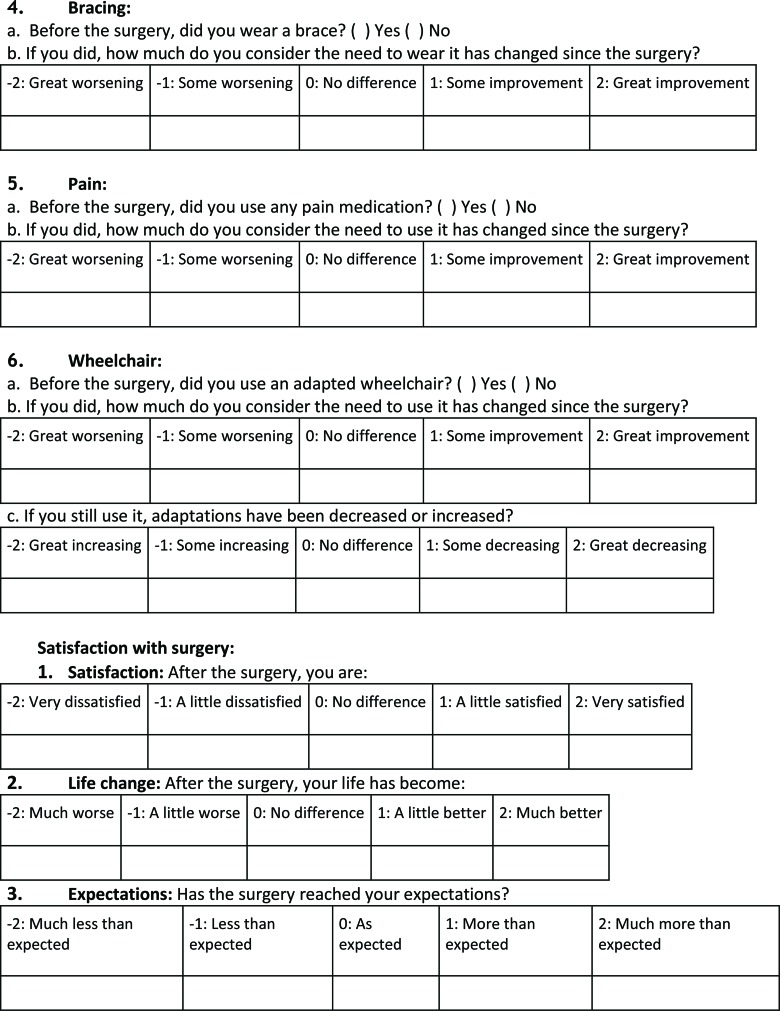

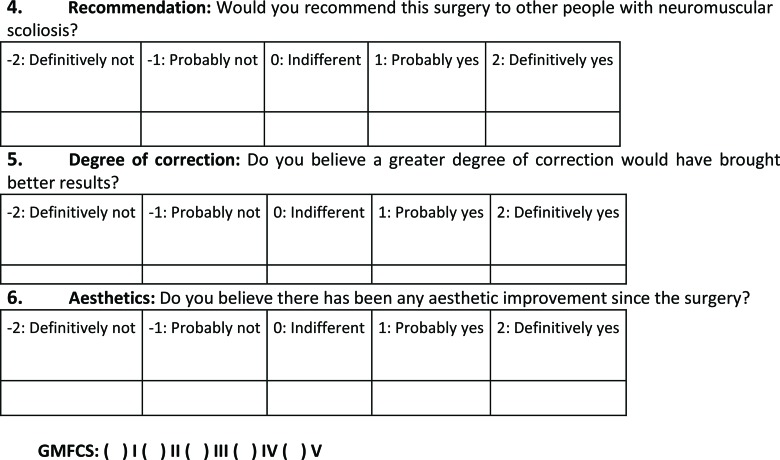
Questionnaire.

A questionnaire adapted from Comstock et al. 10 and Bridwell et al. 15 was created based on the difficulties faced by patients and caregivers (Figure 1). The questionnaire contained 54 questions addressing socio-demographic data (16 questions) and 3 areas: functional ability evaluation, comorbidities, and satisfaction with surgery. The responses to all of the fields were on a graduated scale: very dissatisfied, dissatisfied, indifferent, satisfied, and very satisfied (where appropriate, terms other than “dissatisfied” and “satisfied” were used).

Initially, 50 patients were selected; however, only 32 had the appropriate radiographic series. Of those 32 patients, 2 had died since the surgery and 18 accepted the invitation to be interviewed either in person or via telephone. Of these 18 interviews, 8 were conducted in person in the outpatient clinics and the remaining 10 were conducted via telephone by a participant researcher. Eleven interviewees were the patient’s mother, 2 were the patient's father, 1 was the patient's aunt, 1 was the patient's grandmother and 3 were the patient him/herself.

### Radiographic analysis

To calculate Cobb’s angle, the traditionally described method was used on panoramic radiographs of the spine 18.

The angles were recorded pre- and post-operatively and compared to identify the percentage of correlation (pre – post / pre × 100) and the absolute value of correction in degrees (pre – post).

### Interview analysis

The questionnaire responses were compiled separately for each domain and divided into two groups: positive (very satisfied and satisfied) and negative (indifferent, dissatisfied and very dissatisfied) results. We considered “indifferent” to be a negative result because we believed that submitting someone to an invasive procedure without bringing him/her any improvement would constitute an unsatisfactory outcome.

### Statistical analysis

All of the data were input into a Microsoft Excel^®^ spreadsheet and stored in the cloud (i.e., Dropbox^®^). Following data collection, the data were exported to a statistical analysis program (SPSS^®^ 23.0). For continuous data, we tested whether the distribution was normal, and for paired data, we used the paired t-test. For the correlation analysis of continuous variables, Pearson correlation analysis was used. For the analysis of associations between categorical variables, we used the chi-square test.

## RESULTS

Of the 18 interviewed patients, 8 (44.44%) were male and 10 (55.6%) were female. The mean age was 15.89±3.46 years, and there was no statistically significant difference in age between patients of different gender (*p*=0.907). The patients’ parents lived together in most cases (12, 66.7%). The father was employed in 11 families (61.1%), and the mother was employed in 5 (27.8%). Fifteen families (83.3%) presented a total income between 1 and 3 times the minimum wage. One hundred percent of families had a piped water supply and a sewer system in their homes.

Seven (38.9%) children were illiterate, and 7 were enrolled in special schools. Only 4 (22.2%) children had reached third level (i.e., more than 12 years of schooling in Brazil). Among the parents, 15 (83.4%) fathers and 15 mothers had completed school up to the first degree (i.e., up to 9 years of schooling), but only 3 (16.6%) parents had reached third degree. Thirteen patients (72.2%) considered themselves very religious.

Among the patients, 10 (55%) had cerebral palsy, 2 (11%) had spinal amyotrophy, 2 (11%) had neurofibromatosis, and 4 (23%) had other syndromes (Table 1).

Regarding GMFCS levels, 55.6% of patients were level 5, 22.2% were level 4, 16.6% were level 3, 11.1% were level 2, and 5.5% were level 1.

A summary of the responses to questions concerning functional capacity and comorbidities before the surgery is provided in Table 2.

The mean duration of in-hospital stay was 9.95±5.8 days, with a minimum of 6 days and a maximum of 26 days. Only 2 (11.1%) patients, both of whom had cerebral palsy, presented complications (pneumonia) during their in-hospital stay. Two (11.1%) had to be re-admitted due to later surgical complications (implant loosening associated with infection in one case and pseudarthrosis and rod breakage in the other). The mean follow-up time was 26.7±13.85 months, with a minimum of 3 months and a maximum of 48 months. The Pearson correlation analysis revealed no statistically significant correlation between duration of hospital stay and the Cobb angles before and after surgery.

In the analysis of Cobb angles, surgery obtained a mean correction of 34.17±17.17 degrees (*p*<0.001), with correction ranging from 7 to 63 degrees (Table 3). With respect to the proportion of scoliotic curve, the mean correction was 42.8±15.5% (14.3–63.6%). There was no statistically significant difference between male and female patients in the severity of scoliotic curve or in the absolute degree of correction.

The associations between the proportion or absolute degree of correction and each of satisfaction with surgery and recommendation of intervention were not statistically significant (*p*>0.05). A total of 17 (94.4%) patients were satisfied with the surgery, and 11 (61.1%) had their expectations met. QOL and aesthetics were improved in 83.4% and 94.4% of patients, respectively. All of the patients would recommend the surgery (Table 4).

Among those patients whose absolute mean correction was greater than that of the others, the surgery presented no impact on or worsened QOL (51.67 degrees *vs*. 30.67 degrees, *p*=0.05). The one patient who reported a worsened QOL was a 24-year-old female. She was admitted for seven days for the procedure and presented no complications during or after surgery. She reported worsening in all domains. The patient’s mother also reported that her daughter was not satisfied with the surgery and that her expectations had not been met; however, the patient reported that she would still recommend the surgery to other patients.

Of the interviewed patients, 61.1%, 55.6%, 38.9%, and 61.1% were satisfied with their ability to get dressed, to feed themselves, to sit on their own, and to perform daily living activities, respectively.

A total of 10 (55.6%), 5 (27.8%), 7 (38.9%) and 10 (5.6%) of the interviewed patients reported that they were satisfied with respect to respiratory problems, digestive problems, pain medication and adapted chair use, respectively.

Two patients reported no change in QOL following surgery (an equally negative score both before and after surgery); both were 17-year-old males. One of these patients reported worsening in almost all domains. He was re-admitted due to rod loosening and infection and had the implant removed. Nevertheless, he was satisfied with the surgery and would recommend it.

The second 17-year-old male patient reported worsening in most of the domains. He presented with no complications and was not re-admitted. He was satisfied with the surgery; however, he reported there had been neither an improvement nor worsening in his QOL and answered “indifferent” to the question concerning met expectations. He also responded that he would recommend the surgery.

With respect to the question, “Do you think a greater degree of correction would bring better results?,” the patients who answered negatively experienced a higher degree of correction than did the other patients in terms of both percentage (57.2% *vs*. 37.3%, *p*=0.01) and absolute degrees (47.6 degrees *vs*. 29.0 degrees, *p*=0.035).

## DISCUSSION

Bohtz et al. 19 interviewed 50 patients with cerebral palsy who underwent surgery for the correction of scoliosis and applied the CPCHILD form 20. Twenty-seven patients were female, and 23 were male, with a mean age of 15 years by the time of surgery. The pre-operative Cobb angle was 78.6 degrees (50-120 degrees), and a mean correction of 64.3% (mean final Cobb angle: 28 degrees) was obtained. Watanabe et al. 11 presented similar findings concerning initial and final scoliosis and degree of correction. They interviewed 84 patients with spastic cerebral palsy using their own questionnaire. The mean pre-operative Cobb angle was 88 degrees (53-141 degrees), and the mean post-operative angle was 39 degrees (5-88 degrees).

Suk et al. 21 interviewed 58 patients and applied the MDSQ form for Duchenne’s muscular dystrophy and the SF-36 form for QOL. Twenty-seven patients had Duchenne’s dystrophy, 15 had spinal muscular atrophy, and 16 had progressive muscular dystrophy. All of them were wheelchair bound, and the cohort included 40 males and 18 females, with a mean age of 15 years. The mean pre-operative Cobb angle was 61.5 degrees, and the mean post-operative angle was 39 degrees.

Our study included 18 patients with different neuromuscular diseases, primarily cerebral palsy (55% of patients). The mean pre-operative Cobb angle was 78.8 degrees, and the mean post-operative angle was 44.6 degrees, which indicated a mean correction of 42.8% (mean: 34.2 degrees). Our findings were similar to those of other studies; however, our degree of correction was lower, which may be due to the unique characteristics of our patients, such as their comorbidity severity, spinal curve rigidity, and intraoperative difficulties. The gender distribution was balanced in our study, and there was no preference for sex-linked syndromes, in contrast to the observations of Suk et al. 21.

The rate of adverse events (pneumonia, post-operative complications) was 22.2%, which is similar to the rates of other studies 22. Basques et al. 22 found that higher levels of ASA classification were associated with longer hospital stays and higher rates of infection and adverse events. ASA classification was not evaluated in this study; however, 75% of the patients who presented complications had long hospital stays (20 days or more). There was no correlation between duration of hospital stay and GMFCS in the present study.

Surgery satisfaction was investigated by Bohtz et al. 19, Watanabe et al. 11 and Obid et al. 23, who found results similar to ours. Of the patients interviewed by Bohtz et al., 91.7% were satisfied with the surgery 19. Watanabe et al. found that 92% of patients were satisfied, 94% reported cosmetic improvement, and 71% reported QOL improvement 11. Obid et al. 23 interviewed 32 patients and used a questionnaire that graded patient agreement with statements such as “I am satisfied with the surgery” from 1 (do not agree) to 4 (completely agree). Satisfaction with surgery was 3.95 points on average, improvement in QOL was 3.35 points, and expectations fulfillment was 3.76 points. These results are similar to ours, in which 94.4% of patients reported satisfaction with surgery and aesthetic improvement and 83.4% reported QOL improvement.

In the present study, the surgery for scoliosis correction satisfied almost all of the patients (94.4%) and met the expectations of most (61.1%). The difference in the findings regarding satisfaction and expectation may be attributed to subjective factors (e.g., the intention to improve further, psychological issues, and intellectual comprehension of the procedure) and possible surgical complications that occurred, even if minor. According to Watanabe et al. 11, patient expectations were more related to interruption of the progression of deformity and prevention of cardiopulmonary problems than to improvements in sitting and aesthetics. These aspects were not evaluated in our study. Watanabe et al. 11 divided their patients into response groups of “more satisfied” and “less satisfied” rather than “dissatisfied”. They found that lower satisfaction was correlated with a higher rate of late complications, a lower extent of curve correction, and greater lumbar hyperlordosis. Bohtz et al. 19 observed that posterior instrumentation improved the QOL of patients and that complications, if adequately treated, did not hinder QOL. Comstock et al. 10 observed surgical dissatisfaction among 15% of patients, which the authors attributed to either a post-surgery rebound effect of worsening scoliosis severity and pelvic obliquity or a small initial correction.

In our study, it is possible that the disparity between patient satisfaction with the surgery and the fulfillment of their expectations arose from unrealistic initial expectations or misunderstanding of the objective of the surgery (e.g., believing that the surgery would halt disease progression, resolve all of the patient’s complications, or allow the patient to recover long-lost functions, such as walking or standing). This disparity was not addressed in our study. However, Suk et al. 21 addressed patient function after surgery in more detail. They found that a lower Cobb angle after surgery was positively correlated with improvements in the ability to use both hands and the ability to remain seated for the whole day without pause (3 months and 1 year after surgery) as well as improvements in body pain (3 months), physical function and physical health (3 months and 1 year after surgery).

Patients in the present study were functionally evaluated with the use of broad questions, such as “Was there any improvement in walking ability after surgery?” Although there was no statistically significant difference in functional ability between Cobb angle pre- and post-operatively or the extent of correction, most of the patients reported improvement in almost all the addressed aspects except the ability to stand and walk on their own and personal hygiene. These exceptions may reflect the effects of disease development and not the effects of surgery. In contrast, surgery had positive effects on the use of pain medication, daily living activities and health problems, as also reported by Bohtz et al. 19.

This study showed that patients with greater deformity correction in degrees reported lower (or no improvement of) QOL (although the level of statistical significance was low). However, patients with greater correction also reported that a superior correction would not lead to a greater improvement in QOL. These patients had large pre-operative spinal curvatures (two of the three patients had curvatures greater than 100 degrees) of severe clinical status and achieved a satisfactory post-operative Cobb angle (below 40 degrees in two of the three patients), although their clinical condition remained severe. Such findings further corroborate our hypothesis that satisfaction is multifactorial and not restricted to a quantitative goal. Rather, satisfaction is based on the interpretations of the caretaker and/or the patient of the patient's pathology and progression, and it may be related to unreasonable expectations concerning deformity correction and its effects.

A possible bias of this study concerns the time of application of the questionnaire, which was performed at various times post-operatively (between three months and four years). Natural disease progression may impact patient and caretaker QOL, masking the benefits initially provided by the surgery. Furthermore, in some cases, especially during early post-operative complications or daily living adaptations, surgical benefits may only be observed in the medium to long term.

An important limitation of this study was the small sample size, which was due to insufficient radiographic images and lack of patient availability for interview. The researchers intend to continue with the study and to include more patients to improve the power and precision of the analyses.

Another study limitation concerns the in-person interviews with a physician researcher. This context might have influenced the answers for reasons such as fear of losing treatment, embarrassment, and intimidation, leading to a bias toward positive answers. In the future, it would be preferable to conduct each interview by phone and/or with a non-physician professional to obtain more objective responses.

Few studies have considered the correlation between the degree of neuromuscular scoliosis correction and the change in patient QOL; therefore, there is no ideal correction that will guarantee a true and definitive improvement in QOL. Although this study presents new data on the topic, the researchers intend to more definitively demonstrate whether the degree of correction impacts patient satisfaction or QOL.

In our case series, the satisfaction of patients who received surgical intervention for neuromuscular scoliosis did not depend directly on the degree of deformity correction, although a trend consistent with this relationship was found.

The relationships between satisfaction and the results of the correctional procedure are complex and multifactorial.

## AUTHOR CONTRIBUTIONS

Nordon DG and Lugão AF were responsible for the interview, data acquisition, bibliographic review and manuscript preparation. Machado LC was responsible for the bibliographic review, statistical analysis and manuscript preparation. Marcon RM, Cristante AF and De Barros Filho TE were responsible for the manuscript preparation and final review. Letaif OB was responsible for the data acquisition, bibliographic review, manuscript preparation and final review of the manuscript.

## Figures and Tables

**Table 1:** Patient characteristics (age, gender, disease, GMFCS and Cobb angles).

Age (Years)	Gender	Disease	GMFCS	Cobb (Pre-op)	Cobb (Post-op)
**18**	Male	Cerebral Palsy	5	72	52
**14**	Female	Cerebral Palsy	5	49	19
**16**	Female	Cerebral Palsy	5	33	12
**17**	Male	Cerebral Palsy	5	68	36
**24**	Female	Cerebral Palsy	3	102	39
**17**	Female	Cerebral Palsy	5	85	31
**13**	Female	Cerebral Palsy/Rett	5	86	54
**16**	Female	Cerebral Palsy/Hydrocephalus	5	91	48
**14**	Female	Cerebral Palsy	1	106	48
**17**	Female	Cerebral Palsy	5	100	81
**17**	Male	Neurofibromatosis	4	49	42
**15**	Female	Neurofibromatosis	4	80	49
**17**	Male	Spinal Amyotrophy	4	123	63
**16**	Male	Spinal Amyotrophy	2	103	59
**11**	Male	Goldenhar Syndrome	4	84	48
**22**	Male	Noonan Syndrome	2	74	58
**12**	Female	Congenital Idiopathic Malformation	3	83	43
**22**	Male	Congenital Myopathy	3	30	21

**Table 2:** Main findings concerning physical disabilities and associated comorbidities.

Question		Answer	
		Yes	No
**Able to walk on your own before surgery**	N	6	12
	%	33.3	66.7
**Able to sit on your own before surgery**	N	9	9
	%	50	50
**Able to lie down on your own before surgery**	N	9	9
	%	50	50
**Able to get up on your own before surgery**	N	8	10
	%	44.4	55.6
**Respiratory distress**	N	11	7
	%	61.1	38.9
**Digestive problems**	N	9	9
	%	50	50
**Orthesis use**	N	1	17
	%	5.6	94.4
**Pain medication**	N	9	9
	%	50	50

**Table 3:** Cobb angle values obtained before and after surgery and measures of correction.

	Measurement			
	Minimum	Maximum	Mean	Standard Deviation
**Pre-operative Cobb angle**	30	123	78.8	25.4
**Post-operative Cobb angle**	12	81	44.6	16.8
**Correction (%)**	14.3	63.6	42.8	15.5
**Correction (degrees)**	7	63	34.2	17.2

**Table 4:** Patient or caretaker satisfaction with the surgery.

Domain: Surgery satisfaction	Answer (%)	
	Yes	No
**Surgery satisfaction**	94.4	5.6
**Expectations were met**	61.1	38.9
**Improvement in quality of life**	83.4	16.6
**Aesthetics improvement**	94.4	5.6
**Would recommend the surgery to other patients**	100	0
**Would a greater correction bring better results**	72.2	27.8

## References

[1] Konieczny MR, Senyurt H, Krauspe R (2013). Epidemiology of adolescent idiopathic scoliosis. J Child Orthop.

[2] Kamerlink JR, Quirno M, Auerbach JD, Milby AH, Windsor L, Dean L (2010). Hospital cost analysis of adolescent idiopathic scoliosis correction surgery in 125 consecutive cases. J Bone Joint Surg Am.

[3] Lachaine J, Moreau A, Beauchemin C, Mathurin K (2012). PSU14 Economic impact of scoliosis in Canada: A RAMQ database analysis. Value in Health.

[4] Madigan RR, Wallace SL (1981). Scoliosis in the institutionalized cerebral palsy population. Spine.

[5] Samilson R, Bechard R (1973). Scoliosis in cerebral palsy: incidence, distribution of curve patterns, natural history, and thoughts on etiology. Curr Pract Orthop Surg.

[6] Harrington PR (1962). Treatment of scoliosis. Correction and internal fixation by spine instrumentation. J Bone Joint Surg Am.

[7] Daher YH, Lonstein JE, Winter RB, Bradford DS (1985). Spinal surgery in spinal muscular atrophy. J Pediatr Orthop.

[8] Brook PD, Kennedy JD, Stern LM, Sutherland AD, Foster BK (1996). Spinal fusion in Duchenne&apos;s muscular dystrophy. J Pediatr Orthop.

[9] Suk SI, Lee CK, Min HJ, Cho KH, Oh JH (1994). Comparison of Cotrel-Dubousset pedicle screws and hooks in the treatment of idiopathic scoliosis. Int Orthop.

[10] Comstock CP, Leach J, Wenger DR (1998). Scoliosis in total-body-involvement cerebral palsy. Spine.

[11] Watanabe K, Lenke LG, Daubs MD, Watanabe K, Bridwell KH, Stobbs G (2009). Is spine deformity surgery in patients with spastic cerebral palsy truly beneficial?: a patient/parent evaluation. Spine.

[12] Brazier JE, Harper R, Jones NM, O'Cathain A, Thomas KJ, Usherwood T (1992). Validating the SF-36 health survey questionnaire: new outcome measure for primary care. BMJ.

[13] Asher M, Min Lai S, Burton D, Manna B (2003). The reliability and concurrent validity of the scoliosis research society-22 patient questionnaire for idiopathic scoliosis. Spine.

[14] Askin GN, Hallett R, Hare N, Webb JK (1997). The outcome of scoliosis surgery in the severely physically handicapped child. An objective and subjective assessment. Spine.

[15] Bridwell KH, Baldus C, Iffrig TM, Lenke LG, Blanke K (1999). Process measures and patient/parent evaluation of surgical management of spinal deformities in patients with progressive flaccid neuromuscular scoliosis (Duchenne’s muscular dystrophy and spinal muscular atrophy). Spine.

[16] Gilson KM, Davis E, Reddihough D, Graham K, Waters E (2014). Quality of life in children with cerebral palsy: implications for practice. J Child Neurol.

[17] Bowen RE, Abel MF, Arlet V, Brown D, Burton DC, D’Ambra P (2012). Outcome assessment in neuromuscular spinal deformity. J Pediatr Orthop.

[18] Cobb J (1948). Outline for the study of scoliosis. Inst Course Lect.

[19] Bohtz C, Meyer-Heim A, Min K (2011). Changes in health-related quality of life after spinal fusion and scoliosis correction in patients with cerebral palsy. J Pediatr Orthop.

[20] Narayanan UG, Fehlings D, Weir S, Knights S, Kiran S, Campbell K (2006). Initial development and validation of the caregiver priorities and child health index of life with disabilities (CPCHILD). Dev Med Child Neurol.

[21] Suk KS, Baek JH, Park JO, Kim H-S, Lee HM, Kwon JW (2015). Postoperative quality of life in patients with progressive neuromuscular scoliosis and their parents. Spine J.

[22] Basques BA, Chung SH, Lukasiewicz AM, Webb ML, Samuel AM, Bohl DD (2015). Predicting short-term morbidity in patients undergoing posterior spinal fusion for neuromuscular scoliosis. Spine.

[23] Obid P, Bevot A, Goll A, Leichtle C, Wülker N, Niemeyer T (2013). Quality of life after surgery for neuromuscular scoliosis. Orthop Rev.

